# Association of Intracranial Plaque Features with the Severity of White Matter Hyperintensities in Middle-Aged and Older Community-Dwelling Adults

**DOI:** 10.3390/biomedicines13102553

**Published:** 2025-10-20

**Authors:** Yangyang Cheng, Lihua Lai, Jieqi Luo, Michael Tin Cheung Ying

**Affiliations:** 1 Department of Health Technology and Informatics, The Hong Kong Polytechnic University, Hong Kong; 22037285r@connect.polyu.hk; 2 Department of Radiology, Shenzhen Third People’s Hospital, Shenzhen 518112, China; lailihua7@163.com

**Keywords:** intracranial atherosclerosis, white matter hyperintensity, HR-MRI

## Abstract

**Background/Objectives**: Despite the reported correlation between white matter hyperintensity (WMH) and intracranial atherosclerosis (ICAS), little is known about the association between intracranial plaque imaging characteristics and the severity of WMH. This study aimed to investigate the relationship between plaque imaging features in the major intracranial large arteries and the severity of WMH by high-resolution magnetic resonance imaging (HR-MRI) in a local community-based cohort. **Methods**: Stroke-free Chinese aged over 45 years old were recruited. Plaque imaging features of intracranial arteries identified in middle cerebral arteries (MCAs), vertebral arteries (VAs), and basilar arteries (BAs) were analyzed. The plaque characteristics were compared between subjects with or without moderate-to-severe WMH (Fazekas score > 2), and their independent association with the severity of WMH was also assessed using multivariate logistic regression analysis. **Results**: In the cohort of 272 subjects (mean age, 63.4 ± 6.8 years old; males, *n* = 118), 24.6% with moderate-to-severe WMH had a significantly higher prevalence of ICAS, eccentric lesions, diffuse thickening pattern, and a heavier plaque burden in the intracranial major arteries compared to those without moderate-to-severe WMH. After adjusting for confounding factors, multivariate logistic regression analysis showed that an eccentric pattern of plaque lesion was independently associated with moderate-to-severe WMH. **Conclusions**: Eccentric lesions in the major intracranial large arteries, but not diffuse thickening patterns, luminal stenosis, and plaque burden, were independently associated with a greater burden of WMHs among middle-aged or older adults. Eccentricity of major intracranial large artery lesions may be a potential imaging marker to assess WMH burden. Understanding the correlation between atherosclerotic patterns and the severity of WMH would aid in early stratifying the future clinical risk of cerebrovascular events and support the development of individualized treatment strategies. Further studies are warranted to investigate its value in predicting future cerebrovascular events.

## 1. Introduction

Cerebral small vessel disease (CSVD), referring to small arteries and veins originating from large intracranial blood vessels or their branches [1], is the most common, progressive vascular disease with no optimal treatment to date [2,3]. CSVD is a common term that groups various neuroimaging changes inferred from prominent white matter hyperintensity (WMH), enlarged perivascular space (ePV), lacunar infarct, cerebral microbleed (cMB), and brain atrophy (BA) [4,5]. These pathological changes are strongly linked to each other and often incidentally found on brain scans.

WMH might be an early precursor in the progression of CSVD [6], and is commonly observed in elderly people with a higher prevalence of 72% in individuals over 50 years old [7,8]. Based on the previous population studies, the prevalence of WMH is higher and varies between 45% and 95%, and increases with age [9,10]. White matter hyperintensities, also known as leukoaraiosis, are subclinical brain injuries that indicate pathologic damage within the brain’s microvasculature, including arteries, arterioles, venules, and capillaries [3]. The pathogenetic etiologies of white matter lesions are intricately influenced by a multitude of complex factors, mainly attributed to age-related and vascular risk-factor-related small vessel disease [11]. Blood–brain barrier damage, hypoperfusion to supply white matter, and neuroinflammation, lead to degeneration of myelinated fibers, which are the main pathogenesis of WMH [3,11]. Moreover, the burden of WMH is especially heavy in China [12], and has been implicated in a wide range of neurological impairments, worsening cognitive decline [13], gait imbalance [14], a twofold higher risk of dementia [15], a threefold increase in stroke risk [16], and even a high risk of death [17,18]. Therefore, given their high prevalence and notable clinical implications, it is important to conduct further research on the non-traditional risk factors associated with the progression of WMH, particularly in the elderly population.

Recently, there has been growing evidence reporting that intracranial large artery atherosclerosis may be correlated with CSVD due to sharing some similar vascular risk factors [19,20]. Many traditional risk factors, such as aging [21], hypertension [22], and smoking, have been identified as significant contributors to WMH burden [23]. Age-related ICAS and WMH predispose the brain to a spectrum of adverse cerebrovascular outcomes, including stroke and vascular cognitive decline [24,25], thereby imposing substantial challenges on the healthcare burden. Intracranial atherosclerosis (ICAS) refers to a sticky substance called plaque deposition in the major intracranial arteries, resulting in vessel wall thickening and luminal stenosis, causing these vessels to be narrowed and blocked [12,26]. ICAS is a leading cause of ischemic stroke with the highest rate of recurrent stroke in Asia [27,28]. Although WMH and ICAS are closely related to some vascular risk factors and play an important role in cerebrovascular events, evidence regarding the precise relationship between these conditions remains insufficient. A recent meta-analysis of observational studies found that carotid atherosclerotic stenosis was correlated with the presence of WMH [29]. Apart from atherosclerotic stenosis in the carotid artery, a positive association between the burden of WMH and medial intracranial arterial calcification in patients with acute ischemic stroke was observed in our previous study [7]. In addition, researchers also revealed that a notable association was identified between intracranial atherosclerotic stenosis, intra-plaque enhancement, and greater burden of WMH in patients with cerebral ischemic events [30,31]. The varied results may stem partly from conventional techniques that depended solely on luminal stenosis and patency, failing to consider other critical features that could elucidate the complex interaction between ICAS and WMH burden [20,32]. Traditional methodologies merely superficially observed basic abnormalities in the intracranial arteries, but inadequately characterized vessel wall pathology, overall plaque burden, and key morphological and remodeling features, which can impede suboptimal understanding of cerebrovascular mechanisms and diagnosis and treatment strategies [33]. Subsequently, the adoption of high-resolution vessel wall MRI (HR-MRI) has made it possible not only to delineate plaque load and morphology but also to identify high-risk characteristics that inform prognostic assessment and treatment strategies [34,35]. However, there is still a lack of conclusive evidence on the relationship between the severity of WMH and specific characteristics of intracranial atherosclerotic plaque in the healthy population [31,36,37]. Several studies were retrospective and constrained by methodological issues, including insufficient statistical power and selection bias, while others did not explore or make full use of quantitative plaque metrics derived from vessel wall imaging [38,39].

To advance clinical implementation and guide future therapeutic approaches, investigations need to move beyond the current scope. Therefore, our current study aimed to investigate the potential correlation between atherosclerotic patterns and the severity of WMH in middle-aged or older adults in a local community by high-resolution magnetic resonance imaging (HR-MRI). Advancements in HR-MRI enable high-precision evaluation of plaque-specific features (positive remodeling, plaque load, eccentricity, stenosis severity, and non-stenotic plaque morphology) to examine their differential effects on WMH. Understanding the correlation between atherosclerotic patterns and the severity of WMH would aid in early stratifying the future clinical risk of cerebrovascular events and support the development of individualized treatment strategies.

## 2. Materials and Methods

**Figure 1:**
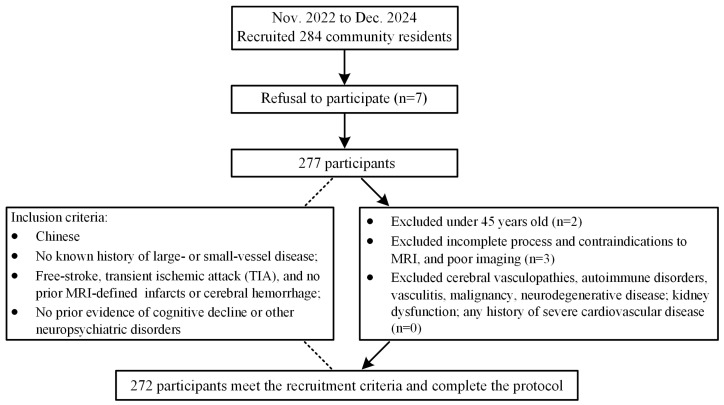
The flowchart shows the subject selection in this study.

The study was approved by the Institutional Review Board of the Hong Kong Polytechnic University (NO. HSEARS20210720002), and all subjects gave written informed consent. Consecutive community inhabitants were recruited from November 2022 to December 2024. Demographic data and clinical information were collected from subjects’ self-reports, electronic medical records, and direct measurements. During the measurement process, vital signs (heart rate, height, blood pressure) and other relevant health metrics were collected by a trained research assistant, thereby ensuring an accurate and comprehensive dataset. The inclusion criteria were as follows: (1) subjects aged over 45 years old, with no known history of large- or small-vessel disease, including no prior stroke, transient ischemic attack (TIA), or recording of prior MRI-defined infarcts or cerebral hemorrhage; (2) no prior evidence of any neurological disorders, such as cognitive decline on the Mini-Mental State Examination (MMSE, a score of <24 is considered potential cognitive impairment) [40] or the Montreal Cognitive Assessment (MoCA) with a score of ≤26 is considered a potential cognitive deficit [41,42]; and (3) subjects underwent HR-MRI scanning and completed the whole process of the study. The exclusion criteria included the following: (1) subjects with clinical signs of cerebrovascular disease, degenerative dementia, or any other neurological impairment identified during the inclusion process were excluded; (2) any contraindication for MRI; (3) other cerebral vasculopathies, including dissection, vasculitis, aneurysm, or moyamoya disease; (4) any history of severe cardiovascular disease, kidney dysfunction (eGFR ≤ 60 mL/min/1.73 m^2^) [43], or malignancy; and (5) poor imaging quality. Table 1: Subject recruitment. This study was reported according to the STROBE guidelines [44]. Figure 1 indicates the subjects’ selection in this study.

The body weight and height of subjects were measured to calculate the body mass index (BMI) using the formula: weight (kg)/height^2^ (m^2^) [45]. Cardiovascular risk factors (hypertension [46], diabetes mellitus [47], hyperlipidaemia [48], etc.), and the use of medication of subjects were recorded.

Brain imaging was performed by a 3.0-T MRI scanner (Siemens Medical Systems, XR Numarism/X VA30A-03GR, Erlangen, Germany) with a 64-channel head-coil. High-resolution vascular sequences (HR-MRI), a transverse 3D T1-weighted volumetric isotopically reconstructed turbo spin echo acquisition; detailed parameters were as follows: field of view: 53 mm × 210 mm × 138 mm, acquired resolution 0.7 mm × 0.7 mm × 0.7 mm, repetition time [TR]/echo time [TE] was 900/15 ms, slice thickness was 0.66 mm. Images were acquired to identify intracranial large-arterial lesions. The standard MRI protocol consisted of 3-dimensional (3D) time-of-flight magnetic resonance angiography (3D TOF-MRA), T2-weighted, T1-weighted, and FLAIR sequences were used to evaluate WMH and stenotic lesions. Detailed imaging parameters were provided in the Supplementary Material as Table S1.

MRI images were viewed on OsiriX DICOM Viewer (Geneva, Switzerland), and quantitative measurements were performed using Vesselmass (Pixmeo SARL, Bernex, Switzerland, version 13.0.2 ) as previously described [49,50,51].

We analyzed all the plaques in the bilateral M1 segments of middle cerebral arteries (MCAs), bilateral intracranial segments of vertebral arteries (VAs), and the basilar artery (BA). Intracranial atherosclerotic lesions were evaluated using 3D HR-Vessel Wall images, and MRI measurements were performed on the derived cross-sectional slice reconstructions. Only the thickest vessel wall was chosen for measurements in each plaque, while the nearest plaque-free cross-section proximal to the plaque was selected as the reference site [35,52]. Quantification of ICAS entailed calculating the OWA (outer wall area) and the LA (lumen area), with data gathered from the lesion itself and a comparable reference segment [53]. The assessment of the plaque imaging features, including plaque burden, was calculated by (plaque area/OWAlesion) × 100% [35]; the remodeling index (RI) was based on the quotient of the lesion’s outer wall area over the reference area (OWAlesion/OWAreference) [54,55]; and other morphological patterns (e.g., focal/diffuse thickening patterns, irregular surface, eccentricity) were mentioned in previous publications [35,56]. Irregular morphology in transects is defined as the presence of disruptions or discontinuities in the plaque surface, while regular morphology is characterized by a smooth and uninterrupted inner wall. In accordance with a previous study, the distribution of the lesion was classified as eccentric if the maximal wall thickness exceeded twice the thinnest wall thickness at the site of maximal lumen narrowing, while it was categorized as concentric if the wall involvement was less than 50% [57,58]. Intracranial lesions were categorized based on their involvement in the thickness scope along the trajectory of intracranial arteries. Lesions covering a longer trajectory (>0.5 cm) were considered to have a diffuse pattern, while lesions confined to a short region (<0.5 cm) or appearing as a dot were classified as having a focal pattern. Then we further assessed the degree of stenosis by a previously described method: (1-lumen area lesion/lumen area reference) × 100% [35]. In light of the absence of well-defined criteria for the precise evaluation of intracranial arterial stenosis severity, and considering that the diameters of major intracranial arteries are slightly narrower and have more branches than extracranial arteries (Figure S1). According to previous reports, vessels with stenotic degrees over 25% on HR-MRI were identified on the matched TOF-MRA, and the stenosis was classified as normal or <25%, 25–49%, ≥50% stenosis [59,60,61].

**Figure 2:**
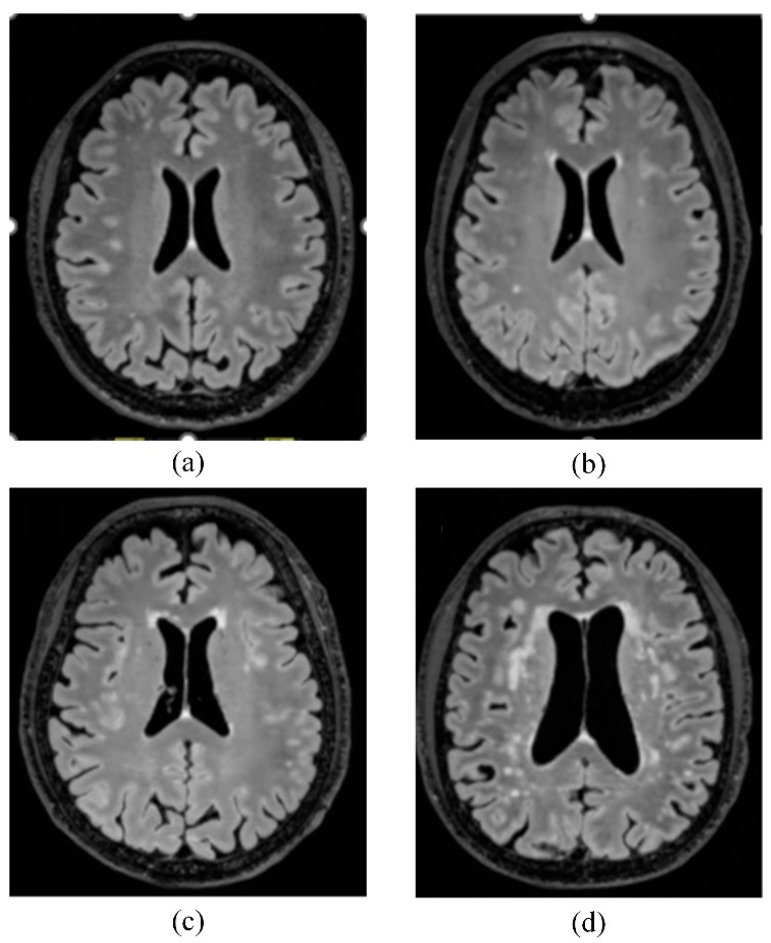
Representative cases of the different degrees of white matter lesions classified by the Fazekas scale on FLAIR. (**a**) Absent WMH, no visible white matter hyperintensities; (**b**) mild WMH, “caps” or pencil-thin lining hyperintensities visualized on periventricular white matter region and punctate foci on deep white matter region; (**c**) moderate WMH, beginning confluence on deep white matter region or smooth “halo” hyperintensities on periventricular white matter region; (**d**) severe WMH, irregular periventricular hyperintensities extending into the deep white matter.

MRI images were independently analyzed by two observers (L.H. L and Y.Y. C) who have at least 3 years of experience in MRI image interpretation, and they were all blind to the clinical data of the participants. In the case of disagreement between the two observers, the images were reviewed by a third observer (J.Q. L), who has more than ten years of neuroimaging experience, and a consensus agreement was made among the three observers. WMH are defined as bright and increased signal on both T2-weighted and fluid-attenuated inversion recovery (FLAIR) MRI, which involves different regions of deep or periventricular sites [62]. The severity of WMH was quantified according to Fazekas et al. rating scale from absent to severe (Figure 2), which is a quick way to estimate functional outcome [7,62,63]. The severity of WMH was evaluated in accordance with a validated Fazekas score scale. The details are below: mild WMHs were defined as a total score of ≤2 (punctate foci in PV-WMH and D-WMH regions), moderate WMHs as a total score of 3–4 (classified as beginning confluence in PV-WMH and D-WMH regions), and severe WMHs as a total score of 5–6 (characterized by large confluent bright signals extending from the PV-WMH area into the deep white matter region) [64].

IBM SPSS version 27.0 was adopted for statistical analyses. All quantitative data were presented as means ± standard deviation (SD) for normally distributed data or as median with interquartile range (IQR) for non-normally distributed data. Categorical variables were presented as numbers and percentages. The baseline characteristics, intracranial plaque features, and the grade of luminal stenosis were compared between subjects with and without moderate-to-severe WMH by independent *t*-test, U test, Pearson’s chi-square test, and Fisher’s exact test when appropriate. Cardiovascular risk factors, including age, hypertension, hyperlipidemia, diabetes, and antithrombotic drugs with *p* < 0.05, were considered as confounding factors and were tested by collinearity diagnostics. To determine the independent association between intracranial plaque characteristics and higher burden of WMHs, univariate and multivariate logistic regression were utilized. The inter-rater reliability of two observers (LH. L and YY. C) was assessed by Cohen’s kappa analysis. A two-sided *p* < 0.05 was considered statistically significant.

## 3. Results

Demographic characteristics of the study population (*n* = 272) are presented in Table 1. Overall, the mean ages were 63.4 ± 6.8 years old, and 118 (43.4%) of the subjects were male. Of the 272 subjects, 67 (24.6%) had moderate-to-severe WMH. Subjects with moderate-to-severe WMH, compared to those with nil–mild WM, were older (66.7 ± 7.5 years old vs. 62.3 ± 6.2 years old, *p* < 0.001), and had significantly greater systolic blood pressure (134.0 ± 14.4 vs. 128.6 ± 17.7, *p* = 0.024), compared to those without moderate-to-severe WMH. Subjects with moderate-to-severe WMH more frequently had vascular risk factors, including hypertension, hyperlipidemia, and diabetes, and a history of cardiac disease (all *p* < 0.050).

The comparison of atherosclerotic plaque characteristics between participants with and without moderate-to-severe WMH is included in Table 2. Among 272 subjects, 152 (55.9%) exhibited different degrees of intracranial atherosclerotic luminal stenosis, and a total of 209 (15.4%, 209/1360) lesions were identified. Participants with moderate-to-severe WMH were more prone to be involved in ICAS (67.2% vs. 52.2%, *p* = 0.032) and more likely to have specific morphological plaque features, including diffuse thickening patterns and eccentricity (all *p* < 0.050). Additionally, a positive correlation was observed between WMH severity and both plaque burden and luminal stenosis, as indicated in the Supplement Material Figure S2. Individuals with moderate-to-severe WMH presented with greater plaque burden and more severe intracranial luminal stenosis compared with those without moderate-to-severe WMH (all *p* < 0.050).

Univariate logistic regression analysis showed that luminal stenosis, diffuse thickening pattern, and eccentricity were correlated with moderate-to-severe WMH (all *p* < 0.050). (Table 3) After covariate adjustment for age, diabetes, hyperlipidemia, sex, smoking, antithrombotic drugs, and hypertension, eccentricity (OR = 1.47; 95% CI 1.04–2.10; *p* = 0.036) was independently associated with moderate-to-severe WMH.

Inter-rater agreement was assessed by randomly selecting 227 subjects. The inter-observer agreements of the characteristics of ICAS were good. Cohen’s kappa of ICAS presence was 0.86 (95% CI 0.793–0.926, *p* < 0.001). Cohen’s kappa of plaque eccentricity was 0.77 (95% CI 0.692–0.847, *p* < 0.001), plaque irregularity was 0.82 (95% CI 0.727–0.912, *p* < 0.001), and diffuse thickening was 0.71 (95% CI 0.631–0.797, *p* < 0.001). Cohen’s kappa of WMH presence was 0.81 (95% CI 0.686–0.933, *p* < 0.001). Cohen’s kappa of classifying WMH severity was 0.78 (95% CI 0.699–0.857, *p* < 0.001) (Table S2).

## 4. Discussion

In this observational study based on a local community, we reported the presence of ICAS identified by HR-MRI and correlated the specific imaging features of asymptomatic intracranial atherosclerotic plaque with the burden of WMH. The results revealed that 55.9% of the individuals in the community-based cohort were identified with non-stenosing, asymptomatic intracranial atherosclerotic lesions, with the majority coexisting with different burdens of WMH. As characterized by HRMRI, luminal stenosis, plaque burden, and certain specific morphological features in the intracranial vessel wall may play a synergistic role in the occurrence and development of WMH. We also expand the importance of the association among plaque imaging phenotypes of intracranial atherosclerosis, as a marker for higher burden of WMH. Recognizing these plaque details can also inform the neurologist before invasive examination to mitigate procedural risks (such as vessel injury or plaque rupture), enhance early-risk screening, and optimize the treatment of WMH.

By far, although recent reports advocate the benefit of intracranial-plaque vessel-wall MRI, there is limited conclusive evidence regarding whether HR-MRI, as a non-invasive imaging modality, provides valuable information for predicting the progression of white matter lesions, and which characteristics are associated with the severity of WMH among many different characteristics in the general population. Hence, in our current study, from a new perspective to explore CSVD, we also pay more attention to finding these potential correlations between atherosclerotic patterns and moderate-to-severe WMH, through detailed analysis of imaging plaque features by HR-MRI. Consistent with our findings, Zhang GS et al. demonstrated that the distribution and morphological plaque features, such as eccentricity and positive remodeling, are closely associated with an increased cerebral WMH burden in symptomatic stroke patients with severe MCA stenosis [64]. The validity of the previous findings is supported by the present community-based design and may provide a more accurate reflection of the association between the presence of intracranial eccentric plaque and the burden of WMH in the aging population, with a broader age range and a more generalizable population. The association of intracranial atherosclerosis with WMH has also been proven by another study in asymptomatic adults, which indicated that greater ICAS severity was associated with a larger WMH volume [65]. We agree that volumetric quantification is more advantageous than a visual Fazekas rating scale. However, volumetric measurement is time consuming, and special software packages are required [66]. Therefore, a notable strength of our study is the adoption of the Fazekas rating scale—a reliable, semi-quantitative, and internationally accepted method [67]. Some previous studies reported that eccentric thickening is frequently used as a criterion to identify ICAS and may be associated with ischemic events [68,69,70]. A clinical study by Ohara T et al. demonstrated that the eccentric plaque is deemed a potential biomarker for ipsilateral cerebrovascular events [69]. In part, our results are consistent with the findings of previous studies, and indicate that the formation of cerebral aortic plaques may serve as a signal reflecting cerebrovascular health and reveal that a certain degree of injury already exists in cerebral small vessels [53,64]. In line with our results, ICAS burden was more likely to present in eccentricity, which might promote aggravation of hypoperfusion to WMH [58,70]. This result could be explained by some plausible reasons: white matter changes may be attributed to alterations in intracranial vascular pulsatility [71], which are thought to be a consequence of progressing arterial stiffness. As ICAS plaques evolve across multiple intracranial vessel beds, they increase global arterial stiffness, may restrict vasodilation, and reduce blood flow. The eccentricity of plaque may remodel the structural changes in vascular dimensions, thereby directly impacting the distribution of cerebral blood flow by exerting its influence on the arterial wall. These geometric alterations and impaired vessel wall compliance lead to increased vascular resistance and compromised blood flow in vast subcortical regions, thereby exacerbating hypoperfusion in the white matter region, particularly when metabolic demand and neuronal activity increase or vasodilatory challenge [7]. In addition, the increased transmission of pulsatile waves, driven by ICAS-induced arterial stiffness, from the aorta into the microvasculature, may pose a significant threat to the fragile cerebral microcirculation [72]. However, some investigators found contrasting findings regarding the association between eccentric plaque and cerebrovascular disease [56,58,73]. Such inconsistency might be caused by different study populations and designs. Based on previous histopathologic evidence, that the geometric presentation of intracranial atherosclerosis—whether eccentric or concentric—is an important morphological feature, but does not correlate with the risk of subsequent brain ischemia [58]. In the past, intracranial atherosclerosis has received less attention compared to extracranial atherosclerosis, such as carotid or aortic stiffness, particularly in stratifying stroke-free elderly populations. This oversight may be attributed to variations in evaluation techniques and the challenges associated with observing cerebral arteries and their latent status using conventional diagnostic imaging modalities [74]. Besides the traditional vascular risk factors such as age, hypertension, diabetes, hypercholesterolemia, and smoking [75,76,77,78], the Rotterdam Study has reported that intima-media thickness (IMT) of the common carotid artery (CCA), carotid plaque burden, and stenosis measured by ultrasonography are associated with WMH [79]. Recently, Heng et al. demonstrated that different patterns of intracranial arterial calcification identified by CT are also associated with the burden of WMH classified by the eight-score criterion in stroke patients [7]. Moreover, a study involving a Korean population indicated that the presence of ICAS was independently associated with WMH severity [36]. Therefore, our findings contribute to addressing a notable gap in the understanding of the association between characteristics of atherosclerotic plaque and WMH development.

On the other hand, Ni et al. found that ICAS contributes to the WMH formation by examining plaque enhancement, luminal stenosis, and cerebral perfusion through comparison of different sites of deep WMH volume. [30]. It has been reported by a 3-year retrospective longitudinal study that the severity of luminal stenosis in the intracranial arteries was positively associated with WMH progression [80]. Whereas we did not observe any significant association between intracranial arterial stenosis and moderate-to-severe WMH, probably due to the majority of subjects having <50% intracranial arterial stenosis on HR-MRI, which was not sufficient to cause a significant cerebral blood hypoperfusion, other specific plaque characteristics have been found to correlate with WMH burden [64]. Moreover, even though atherosclerosis-induced arterial stenosis may influence the perfusion in small blood vessels, resulting in ischemic reactivity to the regions of white matter tracts [81], the absence of an association between intracranial arterial stenosis and moderate-to-severe WMH might be attributed to compensatory mechanisms such as collateral circulation [82,83]. Furthermore, we did not find any evidence to support the significant association between plaque irregular surface, diffuse thickening, and moderate-to-severe WMH. This may be due to the relatively small sample size. To mitigate such bias and find stronger associations between ICAS and WMH, future research may be needed to increase sample size and multi-center collaboration for a comprehensive investigation. The results of the present study contribute significant additional value to the understanding of WMH from multiple perspectives, enriching the knowledge surrounding this complex and uncertain area of research. The present results identified a vulnerable imaging marker for ICAS, and this feature is closely associated with the development of leukoaraiosis. As such, these valuable findings and potential mechanisms establish a robust theoretical framework for future investigations aimed at exploring the clinical benefits of intracranial vessel-wall MRI, including the monitoring of patients with ICAS and the assessment of their response to therapeutic interventions. Moreover, cerebral white matter lesions are commonly observed in the elderly population without any apparent neurological syndrome. To our knowledge, WMH, as a surrogate of CSVD, is strongly associated with advanced age, systolic blood pressure, hyperlipidemia, and other vascular risk factors, which have been consistent with the present study [36,84,85]. Even though the mechanism of leukoaraiosis remains unclear, these risk factors potentially contribute to the development of white matter progression, involving irregular cerebral blood flow, endothelial dysfunction, and inflammation, which disrupt the blood–brain barrier and lead to demyelination and gliosis, and fibrinoid necrosis in the region of white matter. Moreover, a growing body of evidence reports that WMH is closely associated with intima-media thickness as well as the number of carotid plaques, which was also verified in previous work [7,36,79]. Our current study further observed that due to cerebral large arteries and small vessels sharing some risk factors, the burden of white matter lesions may have a graded correlation with the severity of intracranial stenotic arteries and specific plaque characteristics. These findings highlight the substantial impact of ICAS patterns on the progression of leukoaraiosis, which extends previous findings in the existing clinical literature regarding the role of ICAS in WMH in a community-based cohort. Therefore, beyond focusing on the interplay of cerebral large and small vessel disease, early HR-MRI-based identification of ICAS plaque features is paramount. This approach not only enables more accurate risk stratification but also directly informs individualized therapeutic strategies.

There are some limitations in our study. Firstly, this is a community-based, stroke-free cohort addressing subclinical disease, the early/subclinical stages, and a relevant and understudied population. However, the lack of external validity and the small severe-stenosis subgroup limit generalization and precision for this study. Secondly, the present study lacks data on kidney function (eGFR/proteinuria) and carotid atherosclerosis features such as intima-media thickness, total plaque area, and maximum stenosis percentage. Future studies may explore whether white matter lesions are influenced by these factors and investigate potential mechanisms. Lastly, we did not investigate the associations between ICAS and other neuroimaging subtypes of cerebral small vessel disease, such as lacunar infarcts, microbleeds, enlarged perivascular spaces, and cerebral atrophy, as well as not address attention to the cognitive decline. Hence, further studies to investigate the association between ICAS and these neuroimaging characteristics, as well as their influence on the subject’s cognitive ability, are suggested.

## 5. Conclusions

Intracranial large-arterial atherosclerotic plaque characteristics are correlated with the severity of WMH in MRI. Intracranial atherosclerotic lesions with eccentric patterns in MRI may be associated with a higher burden of WMH. Particularly, plaque eccentricity is an independent risk factor of moderate-to-severe WMH. The current study provides an important new direction to explore the association between eccentricity of plaque and progressively greater WMH burden in healthy subjects by evaluating plaque imaging features by HR-MRI. Diagnostic imaging characteristics and WMH can serve as clinical risk predictors contributing to the prediction of clinical prognosis and providing appropriate pre-symptomatic treatment design.

## Figures and Tables

**Table 1:** The comparisons of clinical characteristics between participants with and without moderate to severe WMH.

Characteristics	Total (*n* = 272)	Adults Without Moderate-to-Severe WMH (*n* = 205)	Adults with Moderate-to-Severe WMH (*n* = 67)	*p*
Demographics				
Age, year, mean ± SD	63.4 ± 6.8	62.3 ± 6.2	66.7 ± 7.5	<0.001
Sex, male, *n* (%)	118 (43.4)	84 (41.0)	34 (50.7)	0.161
SBP, mmHg, mean ± SD	129.9 ± 17.1	128.6 ± 17.7	134.0 ± 14.4	0.024
DBP, mmHg, mean ± SD	79.9 ± 10.5	79.5 ± 10.7	81.1 ± 9.8	0.275
Body mass index ≥ 25 kg/m^2^, *n* (%)	87 (32.0)	63 (30.7)	24 (35.8)	0.438
Medical comorbidities, *n* (%)				
Hypertension	80 (29.4)	48 (23.4)	32 (47.8)	<0.001
Diabetes mellitus	33 (12.1)	20 (9.8)	13 (19.4)	0.036
Coronary artery disease	17 (6.3)	9 (4.4)	8 (11.9)	0.039
Hyperlipidemia	104 (38.2)	69 (33.7)	35 (52.2)	0.007
Medications, *n* (%)				
Statins	84 (30.9)	58 (28.3)	26 (38.8)	0.090
Antiplatelet	13 (4.8)	8 (3.9)	5 (7.5)	0.316
Anticoagulant	31 (11.4)	19 (9.3)	12 (17.9)	0.048
Habits, *n* (%)				
Current Smoker	14 (5.1)	10 (4.9)	4 (6.0)	0.752
Alcohol Drinking	20 (7.4)	15 (7.3)	5 (7.5)	0.968

SBP, Systolic blood pressure; DBP, Diastolic blood pressure; WMH, White matter hyperintensity.

**Table 2:** The comparison of atherosclerotic plaque characteristics between participants with and without moderate-to-severe WMH.

	Total (*n* = 272)	Adults Without Moderate-to-Severe WMH (*n* = 205)	Adults with Moderate-to-Severe WMH (*n* = 67)	*p*
The presence of ICAS, *n* (%)	152 (55.9)	107 (52.2)	45 (67.2)	0.032
ICAS in MCA, *n* (%)	105 (38.6)	70 (34.1)	35 (52.2)	0.008
ICAS in VA, *n* (%)	68 (25.0)	46 (22.4)	22 (32.8)	0.088
ICAS in BA, *n* (%)	36 (13.2)	21 (10.2)	15 (22.4)	0.011
The degree of stenosis, *n* (%)				0.072
<25%	154 (56.6)	121 (59.0)	33(49.3)	
25–49%	95 (34.9)	71 (34.6)	24 (35.8)	
≥50%	23 (8.5)	13 (6.3)	10 (14.9)	
Morphological features				
Irregular surface (*n*, %)	65 (23.9)	46 (22.4)	19 (28.4)	0.110
Diffuse lesion (*n*, %)	110 (40.4)	79 (38.5)	31 (46.3)	0.032
Eccentric lesion (*n*, %)	119 (43.8)	82 (40.0)	37 (55.2)	0.029
Positive remodeling index (*n*, %)	94 (34.6)	64 (31.2)	30 (44.8)	0.087

ICAS, Intracranial atherosclerosis; MCA, Middle cerebral artery; VA, Vertebral artery; BA, Basilar artery; WMH, White matter hyperintensity.

**Table 3:** The association between the severity of WMH and the imaging features of ICAS.

	The Severity of WMH Odds Ratio (95%CI)	*p*
Characteristics		
Stenosis degree (%)	1.52 (1.01–2.30)	0.046
Plaque burden (%)	1.01 (1.00–1.02)	0.055
Irregular surface	0.74 (0.54–1.01)	0.060
Diffuse thickening pattern	1.41 (1.01–1.96)	0.041
Eccentric lesion	1.59 (1.13–2.22)	0.007
Model-2 Adjusted for confounding factors (age, hypertension, diabetes, hyperlipidemia, sex, smoking, and antithrombotic drugs)		
Stenosis degree (%)	1.28 (0.82–2.02)	0.281
Plaque burden (%)	1.01 (0.99–1.01)	0.259
Irregular surface	0.87 (0.62–1.22)	0.418
Diffuse thickening pattern	1.19 (0.83–1.71)	0.334
Eccentric lesion	1.47 (1.04–2.10)	0.036

*p* < 0.05; OR, Odds ratio; 95% CI, 95% confidence interval.

## Data Availability

The datasets generated and/or analyzed during the current study are available from the corresponding author (Michael Ying) upon reasonable request.

## Supplementary Materials

The following supporting information can be downloaded at: https://www.mdpi.com/article/10.3390/biomedicines13102553/s1, Figure S1: Eccentric thickening pattern on HR-MRI; Figure S2: Comparison of WMH severity in relation to plaque burden and luminal stenosis. Table S1: Parameters for conventional MRI sequences; Table S2: The inter-observer reliability of analyzing the presence of intracranial atherosclerosis (ICAS) with other plaque imaging features and WMH severity.
