# Supplementary material for: Association of Intracranial Plaque Features with the Severity of White Matter Hyperintensities in Middle-Aged and Older Community-Dwelling Adults
Source: Biomedicines. 2025 Oct 20;13(10):2553. doi: 10.3390/biomedicines13102553 (PMC12562004; doi:10.3390/biomedicines13102553)
Supplement: Supplementary file 1 [file biomedicines-13-02553-s001.zip › biomedicines-3829597-supplementary.pdf]

Supplementary materials

Table S1. Parameters for conventional MRI sequences.

Sequences	T1W I	T2WI	FLAIR	TOF-MRA
**TR, ms**	2500	4100–6000	7000	20.3
**TE, ms**	2.22	88–99	395	4.3
**Acquired resolution, mm^3**	0.8×0.8×0.8	/	0.9×0.9×0.9	0.5×0.5×0.3
**Field of view, mm^3**	240×256×167	263×350×350	230 × 230× 173	263 × 350× 350
**Slice thickness, mm**	0.8	4.0	0.9	/

Table S2. The inter-observer reliability of analyzing the presence of intracranial atherosclerosis (ICAS) with other plaque imaging features and WMH severity

	Observer 1	Observer 2	Inter-observer agreement Coefficient (95% CI)
Presence of ICAS (MCA, BA, VA); (n, %)	127 (55.9)	123 (54.2)	0.86 (0.793 – 0.926)
Plaque eccentricity; (n, %)	102 (45%)	86 (37.9%)	0.77 (0.692 – 0.847)
Plaque irregular surface; (n, %)	51 (22.5)	45 (20.0%)	0.82 (0.727 – 0.912)
Plaque diffuse thickening (n, %)	94 (41.4%)	78 (34.4%)	0.71(0.631 – 0.797)
Presence of WMHs	207 (91.2%)	199 (87.6%)	0.81 (0.686 – 0.933)
WMHs grade			0.78 (0.699 – 0.857)
Mild	144 (63.4%)	132 (58.1%)	
Mod	59 (26%)	60 (26.4%)	
Severe	4 (2%)	7 (3%)	

p<0.05; OR, Odds ratio; 95% CI, 95% confidence interval.

Figure S1. Eccentric thickening pattern on HR-MRI.

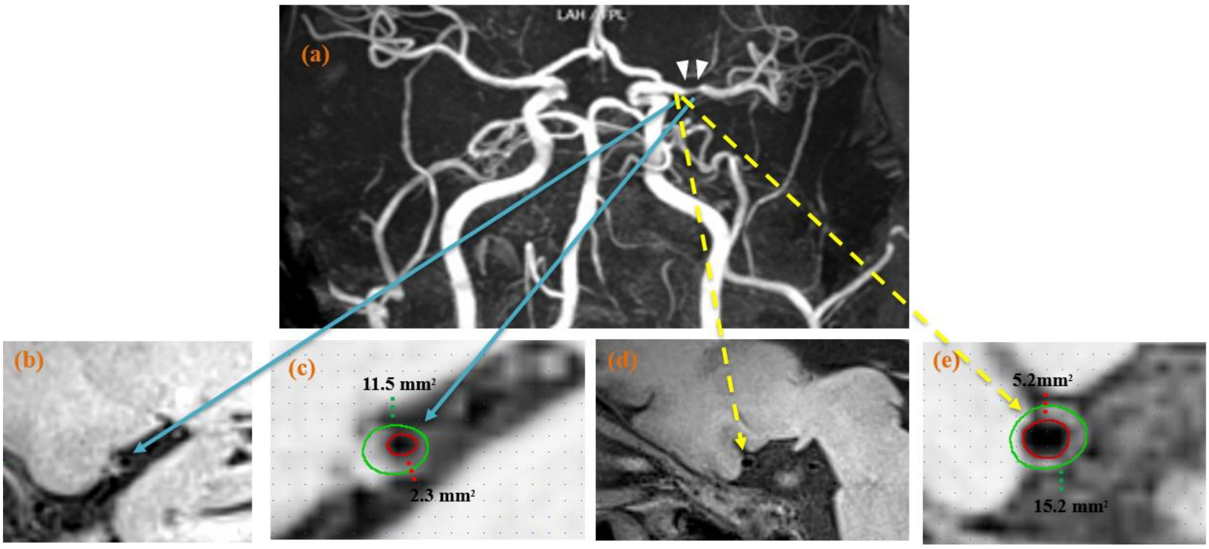

(**a**–**e**) shows an example of eccentricity of intracranial plaque in subject with moderate to severe luminal stenosis of left MCA (M1). (**a**). Left MCA (M1) with significant luminal narrowing on MRA (white arrow). T1-weighted sagittal images, reconstructed from 3D high-resolution MRI acquisition, were utilized for lesion analysis (**b, c**) and reference site analysis (**d, e**). Contours were drawn to delineate the outer wall of vessel (green) and lumen (red) of the nearest plaque-free or minimum lesion segment proximal to the lesion, which was selected as reference sites (**d**), and the cross-section with the thickest plaque was selected as the maximum luminal narrowing (MLN) site (**c**). The eccentricity was computed as = (11.5 − 2.3)/11.5 = 0.80 > 0.50, indicative of eccentric plaque.

Figure S2. Comparison of WMH severity in relation to plaque burden and luminal stenosis.

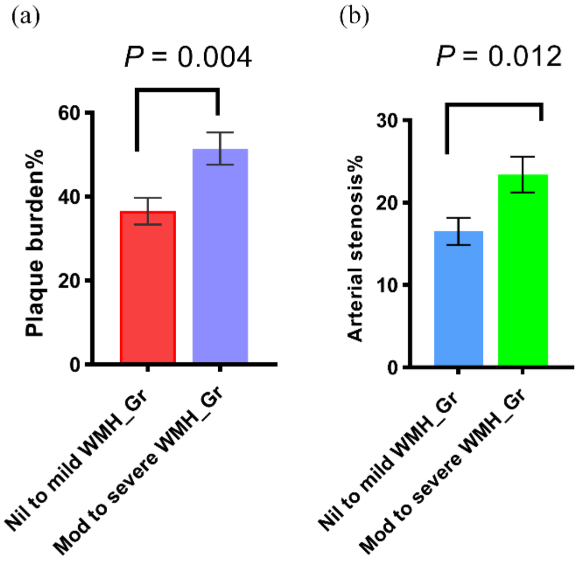

(**a**) Individuals with moderate-to-severe WMH were presented with greater plaque burden. (**b**) Individuals with moderate-to-severe WMH were more likely to appear severe intracranial luminal stenosis.
